# Morinda Officinalis Oligosaccharides Alleviate Post‐Stroke Depression‐Like Behaviors Alongside Increased Gut Microbial Diversity and Butyrate Levels and Reduced Hippocampal Pla2g4a Expression

**DOI:** 10.1002/cns.71238

**Published:** 2026-10-30

**Authors:** Hui Ma, Yue Chen, Xinzhou Deng, Xuan Fang, Wenhui Xiao, Qian Long, Lu Du, Fuze Du, Haonan Chen, Yan Wang, Bo Gao, Xin Qiao

**Affiliations:** ^1^ Academy of Military Medical Sciences Academy of Military Sciences Beijing China; ^2^ Graduate Collaborative Training Base of Academy of Military Medical Sciences, Hengyang Medical School University of South China Hengyang China; ^3^ Chinese PLA Medical School Beijing China; ^4^ Anhui Medical University Hefei China

**Keywords:** butyrate, hippocampus, intestinal and blood–brain barrier, MOO, Pla2g4a, PSD

## Abstract

**Background and Purpose:**

Post‐stroke depression (PSD) represents a common and debilitating complication after cerebral ischemia, yet mechanism‐based therapies remain an urgent unmet need. The microbiota‐gut‐brain axis has been implicated in PSD pathophysiology, but the precise molecular pathways are largely elusive. Morinda officinalis oligosaccharides (MOO), a clinically approved antidepressant in China, exhibit poor oral absorption, raising the possibility of a gut‐dependent mechanism.

**Experimental Approach:**

Male C57BL/6J mice were subjected to transient middle cerebral artery occlusion (MCAO) followed by 21 days of chronic restraint stress (CRS) to establish a PSD model. MOO (50 mg/kg/day) was administered via gavage from Day 7 to 27. Depression‐like behaviors, 16S rRNA‐based gut microbiota profiling, short‐chain fatty acid (SCFA) quantification, intestinal and blood–brain barrier integrity assessments, and hippocampal proteomics were performed. The transferability of MOO‐associated fecal effects was assessed using a donor‐controlled fecal microbiota transplantation (FMT) design. The butyrate‐Pla2g4a relationship was further examined by sodium butyrate supplementation and AAV‐mediated hippocampal Pla2g4a overexpression.

**Key Results:**

MOO administration altered gut microbial composition, increased the abundance of probiotics and decreased the abundance of harmful bacteria, and was accompanied by increased fecal butyrate levels. The donor‐controlled FMT experiment provided preliminary evidence that fecally transferable factors from MOO‐treated PSD donors may contribute to behavioral improvements in PSD recipients. MOO and sodium butyrate treatment were associated with improved indices of intestinal and blood–brain barrier integrity. Hippocampal proteomics identified Pla2g4a, a phospholipase linked to neuroinflammation, as a candidate molecule related to the observed effects. Both MOO and sodium butyrate treatment were associated with reduced hippocampal Pla2g4a expression, and Pla2g4a expression was inversely correlated with butyrate levels. Hippocampal Pla2g4a overexpression attenuated the behavioral improvements associated with sodium butyrate treatment, supporting the functional involvement of Pla2g4a in the response to sodium butyrate.

**Conclusions and Implications:**

MOO treatment was associated with improved depression‐like behaviors in PSD mice, accompanied by gut microbial alterations, increased fecal butyrate, improved barrier integrity, and reduced hippocampal Pla2g4a expression. FMT findings provided preliminary evidence that fecally transferable factors from MOO‐treated donors may contribute to behavioral improvements, while Pla2g4a overexpression attenuated the behavioral effects of sodium butyrate. These findings highlight a potential association among MOO, gut‐derived butyrate, barrier integrity, and hippocampal Pla2g4a in PSD.

## Graphical abstract

The mechanism by which MOO alleviates PSD in mice involves remodeling the gut microbiota to increase butyrate, thereby repairing the intestinal barrier and the blood–brain barrier. MOO and butyrate downregulate the hippocampal neuroinflammatory target Pla2g4a, while hippocampal overexpression of Pla2g4a counteracts the antidepressant effects of butyrate.

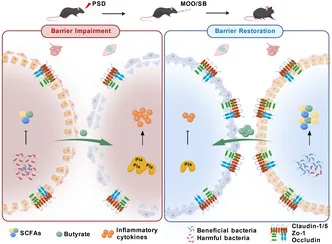

## 1 Introduction

Despite the well‐established adverse effects of PSD, its clinical treatment rates remain suboptimal [1]. Recently, the proposal and refinement of the microbiota‐gut‐brain axis (MGBA) theory have drawn increasing attention to the role of gut microbiota in the pathogenesis and progression of PSD [2]. Emerging evidence reveals that cerebral insults following stroke can trigger systemic stress responses, which alter autonomic nervous tone and compromise intestinal barrier integrity, ultimately leading to gut microbial dysbiosis [3]. This dysbiosis is typically characterized by a reduced abundance of SCFA‐producing bacteria, such as butyrate (C4) producers, and an expansion of pro‐inflammatory microbial populations [4]. Such ecological imbalance exacerbates PSD pathology through multiple circuit mechanisms: First, within the neuroimmune pathway, metabolites from dysbiotic microbiota or their components, such as lipopolysaccharide (LPS), may translocate into the systemic circulation, activating both peripheral and central immune cells and driving a neuroinflammatory cascade that directly impairs neuroplasticity in emotion‐related brain regions, including the hippocampus [5, 6]. Second, at the neuroendocrine level, gut microbiota disruption can impair the negative feedback regulation of the hypothalamic–pituitary–adrenal (HPA) axis, leading to sustained elevation of corticosterone levels and aggravated neuronal damage [7]. Third, through metabolic pathways, microbiota‐derived neuroactive substances (such as tryptophan metabolites) not only influence the synthesis of monoamine neurotransmitters such as serotonin but also interact with receptors abundantly distributed on vagal afferent fibers, establishing direct gut‐to‐brain communication and forming the anatomical basis for bidirectional gut‐brain signaling [8, 9]. Collectively, these mechanistic insights highlight the substantial translational potential of intervention strategies targeting the gut microbiota.

Morinda officinalis oligosaccharides (MOO), natural active components extracted from the traditional Chinese herb Morinda officinalis, have shown promising therapeutic potential for PSD [10]. MOO has been approved as a prescription antidepressant by the National Medical Products Administration (NMPA) of China since 2012 (approval number: Z20120013), with well‐established safety and clinical efficacy in treating mild‐to‐moderate depression. Notably, MOO capsules showed therapeutic efficacy non‐inferior to fluoxetine in a large cohort of 1384 patients with mild‐to‐moderate depression. Despite these clinical benefits, MOO exhibits poor oral absorption, resulting in very low concentrations in the blood or brain [11]. This pharmacokinetic profile suggests that gut‐associated mechanisms may contribute to MOO's antidepressant action. Although previous studies have linked MOO treatment to changes in gut microbial composition [12], how these changes relate to its behavioral effects in PSD remains incompletely understood. Two questions therefore warrant investigation. First, are the behavioral improvements associated with MOO treatment in PSD mice accompanied by changes in gut microbial composition and fecal butyrate levels? Second, can sodium butyrate supplementation improve PSD‐related behavioral and barrier‐associated measures, and is hippocampal Pla2g4a functionally involved in its behavioral effects? Addressing these questions may help clarify the relationships among MOO treatment, gut microbial alterations, butyrate, and hippocampal molecular responses in PSD.

In this study, we integrated neuropharmacology, 16S rRNA‐based microbiome analysis, SCFA profiling, intestinal and blood–brain barrier assessments, hippocampal proteomics, sodium butyrate supplementation, and AAV‐mediated hippocampal Pla2g4a overexpression to evaluate the associations among MOO treatment, microbial remodeling, SCFA changes, barrier‐related measures, and central molecular responses. A donor‐controlled FMT experiment was used to assess whether fecal factors associated with MOO treatment were transferable. This study provides a framework for subsequent mechanistic investigation of microbiota‐associated interventions for PSD.

## 2 Methods

### 2.1 Animals

Male C57BL/6J mice (6–8 weeks old) were obtained, randomly assigned to experimental groups, and maintained under standard specific pathogen‐free conditions (22°C ± 2°C, 12‐h light/dark cycle) with food and water available ad libitum. All animal procedures were conducted in accordance with the relevant institutional guidelines and were approved by the appropriate animal ethics committee.

### 2.2 Materials

MOO (batch No. 2021B03527) was provided by Beijing Tongrentang Co. Ltd. (Beijing, China). The chemical constituents were identified by chemical and spectrometric methods, and four oligosaccharide components were characterized as nystose (tetrasaccharide), 1‐β‐D‐fructofuranosylnystose (pentasaccharide), inulin‐type oligosaccharide hexamer (hexasaccharide), and inulin‐type oligosaccharide heptamer (heptasaccharide). The quality control standard for MOO was as follows: the total content of oligosaccharides with degrees of polymerization ranging from 3 to 9 was 53%–70%, with the pentasaccharide content exceeding 6%. MOO was dissolved in sterile physiological saline and administered by oral gavage at 50 mg/kg [13] to PSD mice starting 1 week after PSD induction, with control mice receiving an equal volume of vehicle.

Sodium butyrate (Sigma‐Aldrich) was freshly dissolved in sterile physiological saline and administered by gavage at a dose of 30 mg/kg/day [14]. The intervention was performed daily from Days 7 to 27 during the PSD modeling period, consistent with the MOO treatment schedule.

### 2.3 Establishment of the PSD Model

The PSD model was established by combining 60‐min MCAO with subsequent CRS. Mice were anesthetized with pentobarbital sodium (50 mg/kg, i.p.), and a monofilament was inserted to occlude the left MCA, followed by reperfusion. Sham‐operated controls underwent the same surgical procedure without filament insertion. After a 1‐week recovery, mice were subjected to daily 4‐h CRS for 3 weeks to induce depression‐like phenotypes.

Mice in the Ctrl group underwent sham surgery, in which the common, internal, and external carotid arteries were exposed and isolated without filament insertion. During the CRS phase, Ctrl mice were not restrained but were deprived of food and water during the same daily period (9:00–13:00) to match the deprivation condition experienced by the PSD groups. Ctrl mice also received an equivalent volume of sterile saline by gavage daily to control for the effects of gavage manipulation.

### 2.4 Antibiotic Pretreatment and Fecal Microbiota Transplantation

To investigate the contribution of the gut microbiota to the effects of MOO, a separate experimental cohort was subjected to the same PSD induction procedure and assigned to four groups: PSD + Veh, PSD + MOO, FMT‐PSD, and FMT‐MOO. The PSD + Veh and PSD + MOO groups served as concurrent non‐FMT comparison groups, whereas the FMT‐PSD and FMT‐MOO groups served as FMT recipients. All mice in these four groups underwent MCAO on Day 0, followed by CRS from Days 7 to 27. Mice in the FMT‐PSD and FMT‐MOO groups received an antibiotic cocktail containing ampicillin (200 mg/kg, Sigma‐Aldrich, A9393), neomycin sulfate (200 mg/kg, Sigma‐Aldrich, N1876), metronidazole (200 mg/kg, Sigma‐Aldrich, BP603), and vancomycin (100 mg/kg, Sigma‐Aldrich, V2002) by oral gavage once daily for five consecutive days (Days 1–5) to reduce the resident gut microbiota. After a 24‐h antibiotic washout period on Day 6, FMT was initiated on Day 7. Mice in the PSD + Veh and PSD + MOO groups received vehicle on Days 1–5, followed by vehicle or MOO (50 mg/kg/day), respectively, by oral gavage on Days 7–27 [15].

Fecal material was obtained from two additional groups of donor mice: vehicle‐treated PSD donors and MOO‐treated PSD donors (*n* = 8 per group). These donors were separate from all four experimental groups; the concurrent PSD + Veh and PSD + MOO comparison groups were not used as donors. The donor protocol was initiated 21 days before the corresponding recipient protocol. Donors underwent MCAO on their own Day 0, followed by CRS and daily oral administration of vehicle or MOO (50 mg/kg/day) on their own Days 7–27. The final day of donor CRS and treatment corresponded to recipient Day 6. Fecal collection and recipient FMT began the following day, corresponding to donor Day 28 and recipient Day 7 [16]. Neither CRS nor vehicle/MOO administration was continued in donors during the subsequent 21‐day fecal collection period. No additional MOO‐specific washout period was imposed before fecal collection.

Fresh fecal pellets were collected individually from all eight donors in each group at the same time of day throughout recipient Days 7–27. For each daily preparation, 25 mg of feces from each donor was combined to obtain a 200‐mg group‐specific pooled sample, ensuring equal contributions by wet weight. Samples from vehicle‐treated and MOO‐treated PSD donors were processed separately. The pooled feces were suspended in sterile normal saline at 100 mg/mL, thoroughly homogenized, and centrifuged at 800 × *g* for 3 min at 4°C to remove large particulate matter. The resulting supernatant was administered to recipients by oral gavage within 10 min of preparation. Mice in the FMT‐PSD and FMT‐MOO groups received 200 μL of the freshly prepared suspension once daily from vehicle‐treated PSD donors and MOO‐treated PSD donors, respectively. Donor microbiota stability after treatment cessation was not assessed, and residual MOO and its metabolites were not quantified in the fecal preparations.

FMT was discontinued after the final administration on Day 27, approximately 24 h before the first behavioral test. The open‐field test, elevated plus‐maze test, tail‐suspension test, and forced‐swim test were performed sequentially on Days 28, 29, 30, and 31, respectively.

### 2.5 Laser Speckle Contrast Imaging (LSCI)

Cerebral blood flow (CBF) was monitored using laser speckle contrast imaging (PeriCam PSI, Perimed). Under pentobarbital anesthesia, mice were positioned in a stereotaxic frame and the skull was exposed. CBF was continuously imaged at 5‐min intervals at baseline and 1 h after MCAO within a fixed 9.42 mm^2^ region of interest and quantified in perfusion units (PU).

### 2.6 2,3,5‐Triphenyltetrazolium Chloride (TTC) Staining

Following a pentobarbital sodium overdose (200 mg/kg, i.p.), brains were harvested and coronally sectioned into 2‐mm slices. Sections were stained with 1% TTC solution at 37°C for 20 min in the dark and then fixed in 4% paraformaldehyde for 24 h. Viable tissue stained red, while infarct areas remained pale/white. TTC staining at 24 h after MCAO was performed in an independent model‐validation cohort; these animals were not used in subsequent behavioral or molecular experiments.

### 2.7 Behavior Assays

#### 2.7.1 Open Field Test (OFT)

After acclimation, mice freely explored an open field arena (40 cm × 40 cm × 20 cm) for 5 min. Total distance and central zone (20 cm × 20 cm) movement were tracked automatically (SMART, RWD). The arena was cleaned with 75% alcohol between trials in a sound‐attenuated room.

#### 2.7.2 Elevated Plus Maze (EPM)

Mice were placed on the central platform of an elevated plus maze (open arms: 30 cm × 5 cm; closed arms: 30 cm × 5 cm × 15 cm) for 5 min. Time spent in the open arms, open‐arm entries, and total arm entries were automatically recorded under quiet conditions.

#### 2.7.3 Tail Suspension Test (TST)

Mice were suspended by the tail in a black chamber for 6 min. Immobility time was scored blindly by two researchers, with discrepancies > 10% resolved by joint video review. Testing was conducted under quiet conditions.

#### 2.7.4 Forced Swimming Test (FST)

Mice were placed in a water‐filled cylinder (45 cm height, 20 cm diameter; 30 cm water depth, 25°C ± 1°C) for 6 min. Immobility (floating with minimal movement for > 5 s) was scored during the last 5 min using the same blinding protocol as in the TST. Tests were conducted between 9:00 and 16:00 under quiet conditions in randomized order.

### 2.8 16S rRNA Sequencing of Fecal Samples

Fecal samples were collected after perianal cleaning with 75% ethanol. Sequencing reads were demultiplexed, adapter‐trimmed, and quality‐filtered. Amplicon sequence variants (ASVs) were generated using DADA2 in QIIME 2, and taxonomic assignment was performed against the SILVA database (confidence ≥ 0.7). Alpha diversity was assessed using the Chao1, Shannon, and Simpson indices. Beta diversity was analyzed by principal coordinate analysis based on Bray‐Curtis distances, and statistical significance was tested using PERMANOVA. Additional multivariate and machine‐learning analyses were used to characterize microbial community features.

### 2.9 SCFAs Detection

Thawed fecal samples were homogenized, and metabolites were extracted with methanol. After centrifugation, the supernatant was derivatized using 3‐nitrophenylhydrazine and EDC in pyridine (30°C, 30 min). Derivatized samples were filtered and analyzed by LC–MS/MS (Waters BEH C8 column; gradient elution with 0.01% formic acid‐water and methanol‐isopropanol) on a Xevo TQ‐micro system under optimized ion‐source conditions.

### 2.10 LC–MS/MS Conditions for Butyrate Detection

Prior to hippocampal collection, mice were perfused with ice‐cold physiological saline to minimize residual blood contamination. The hippocampal tissues were then rapidly collected, and the sample amount was normalized according to tissue wet weight. Butyrate in mouse hippocampal tissue, serum, and fecal samples was quantitatively determined by liquid chromatography–tandem mass spectrometry (LC–MS/MS) following sample pretreatment and derivatization [17]. Chromatographic separation was performed using an ExionLC‐AE system (AB SCIEX, Framingham, MA, USA) equipped with a Kinetex C8 column (100 Å, 2.6 μm, 2.1 mm × 100 mm). Mobile phase A consisted of water containing 0.1% formic acid, while mobile phase B consisted of 50% methanol‐isopropanol containing 0.1% formic acid. The gradient elution program was as follows: 0–1 min, A/B = 80:20 (v/v); 6 min, A/B = 60:40 (v/v); and 9–10 min, A/B = 80:20 (v/v). The flow rate was 0.3 mL/min, the column temperature was maintained at 40°C, the autosampler temperature was set at 4°C, and the injection volume was 1 μL.

Mass spectrometric analysis was performed using a SCIEX QTRAP 6500+ mass spectrometer (AB SCIEX, Framingham, MA, USA) equipped with an electrospray ionization (ESI) source. Data were acquired in negative ion mode using multiple reaction monitoring (MRM). The ion spray voltage was set at −4500 V, and the ion source temperature was 550°C. The nebulizer gas (GS1) and auxiliary gas (GS2) were both set at 50 psi; the curtain gas (CUR) was set at 30 psi, and the collision gas (CAD) was set at 10. Data acquisition and quantitative analysis were performed using SCIEX OS software (version 4.7), and butyrate concentrations in the samples were calculated based on the standard calibration curve.

### 2.11 Immunofluorescence Staining (IF)

Tissue sections were blocked and incubated overnight at 4°C with primary antibodies against tight junction proteins: E‐cadherin (Abcam, ab205336), Claudin‐1 (Invitrogen, PA1‐37465), ZO‐1 (Cell Signaling Technology, 13663S), Occludin (Invitrogen, 40‐4700), Claudin‐5 (Invitrogen, 34‐1600), GFAP (Millipore, MAB360), Iba1 (Abcam, ab178846), CaMKIIα (SANTA CRUZ, sc‐13,141), and Pla2g4a (Cell Signaling Technology, 5249T). After incubation with fluorescent secondary antibodies, nuclei were stained with DAPI. Images were acquired using an FV3000 confocal microscope and analyzed with ImageJ in a double‐blind manner. At least three sections per animal were quantified.

### 2.12 Western Blotting (WB)

Tissue samples were homogenized in lysis buffer and centrifuged to collect the supernatant. Protein concentration was determined using a BCA kit. After separation by SDS‐PAGE and transfer to PVDF membranes, blots were probed overnight at 4°C with primary antibodies against Claudin‐1 (Invitrogen, PA1‐37465), ZO‐1 (Cell Signaling Technology, 13663S), Occludin (Invitrogen, 40‐4700), Claudin‐5 (Invitrogen, 34‐1600), Pla2g4a (Cell Signaling Technology, 5249T), β‐actin (Abcam, ab8227), and GAPDH (Abcam, ab181602). Following incubation with secondary antibodies, bands were visualized and quantified with a gel imaging system. Target protein levels were normalized to β‐actin or GAPDH using ImageJ.

### 2.13 ELISA

Hippocampal tissues were collected from each experimental group, homogenized, and centrifuged to obtain supernatants for subsequent analysis. The concentrations of TNF‐α, LPS, and IL‐6 in the supernatants were quantified using commercial ELISA kits according to the manufacturers' instructions. The optical density (OD) of each well was measured at 450 nm using a microplate reader. Analyte concentrations were calculated from the corresponding standard curves, with sample dilution factors taken into account. All assays were performed with technical replicates to assess measurement consistency.

### 2.14 Stereotaxic Microinjection

Mice were immobilized using stereotactic frames after induction of anesthesia. An AAV‐CMV‐Pla2g4a‐3xFLAG‐EF1a‐EGFP‐tWPA vector (titer: 6.37 × 10^12^ VG/mL; Obio Technology Co. Ltd.) was stereotaxically injected into the bilateral hippocampi (AP = −2.05 mm, ML = ±1 mm, DV = −2 mm from bregma). A total volume of 0.3 μL was delivered per side at a rate of 100 nL/min using a microsyringe pump. The needle was left in place for 5 min and then carefully withdrawn. Mice were allowed to recover on a 37°C heating pad, were monitored for 24 h, and were given buprenorphine (0.05 mg/kg, s.c.) postoperatively for analgesia. For validation of AAV‐mediated Pla2g4a overexpression, hippocampal tissues were collected 21 days after AAV injection.

### 2.15 Sholl Analysis

Astrocyte morphological complexity was assessed via Sholl analysis using ImageJ. Maximum intensity projections from GFAP immunofluorescence Z‐stacks were analyzed by overlaying concentric circles at 2 μm intervals from the soma outward. Intersections between circles and GFAP‐positive processes were quantified at each radius.

### 2.16 Proteomics Data Analysis

Proteomic data were analyzed using MaxQuant with tryptic digestion, cysteine carbamidomethylation, and oxidation/acetylation as variable modifications. Database searches were performed against UniProt. After filtering and contaminant removal, log_2_‐transformed LFQ intensities were imputed for missing values. Data reproducibility was assessed by PCA. Differential protein abundance was analyzed among the Ctrl, PSD, and MOO groups. Results were visualized using hierarchical clustering and heatmaps, followed by GO and KEGG enrichment analysis.

### 2.17 Statistical Analysis

All data are presented as the mean ± standard error of the mean (SEM), unless otherwise indicated. Statistical analyses were performed using GraphPad Prism version 6.0 and the bioinformatics tools described above. Two‐group comparisons were performed using unpaired *t*‐tests. Multiple‐group comparisons were performed using one‐way ANOVA followed by Tukey's post hoc test or the Kruskal–Wallis test followed by Dunn's multiple comparisons test, as appropriate. Two‐way ANOVA was used where indicated in the figure legends. Correlation analyses were performed to assess associations between variables. Differences in microbial community composition were assessed using permutational multivariate analysis of variance (PERMANOVA). A *p*‐value of less than 0.05 was considered statistically significant.

## 3 Results

### 3.1 MOO Ameliorates Depressive‐Like Behaviors in PSD Mice and Partially Restores Gut Microbial Homeostasis

This study employed a mouse model of PSD to simulate the pathogenesis and pathological changes of the corresponding human disorder [18]. The antidepressant agent MOO (50 mg/kg/day) was administered daily from Day 7 to Day 27 during the PSD modeling period to evaluate its effects on depressive‐like behaviors (Figures 1A and S1A). LSCI revealed a significant decrease in regional CBF (rCBF) 1 h after MCAO. TTC staining in an independent model‐validation cohort showed cerebral infarction 24 h after MCAO (Figures 1B and S1B,C), confirming successful induction of ischemic injury but not demonstrating equivalent baseline infarct volumes in the animals subsequently assigned to treatment groups. In the OFT and EPM tests, compared with the Ctrl group, PSD mice exhibited significantly reduced time spent in the central zone and in the open arms, respectively. MOO treatment reversed these deficits. In the TST and FST, immobility times were significantly increased in the PSD group. Both MOO and Flx (the positive control) significantly reduced immobility time compared with the PSD group (Figure 1C,D). These results indicate that MOO treatment was associated with improvement in ischemic stroke‐induced depressive‐like behaviors.

**FIGURE 1:**
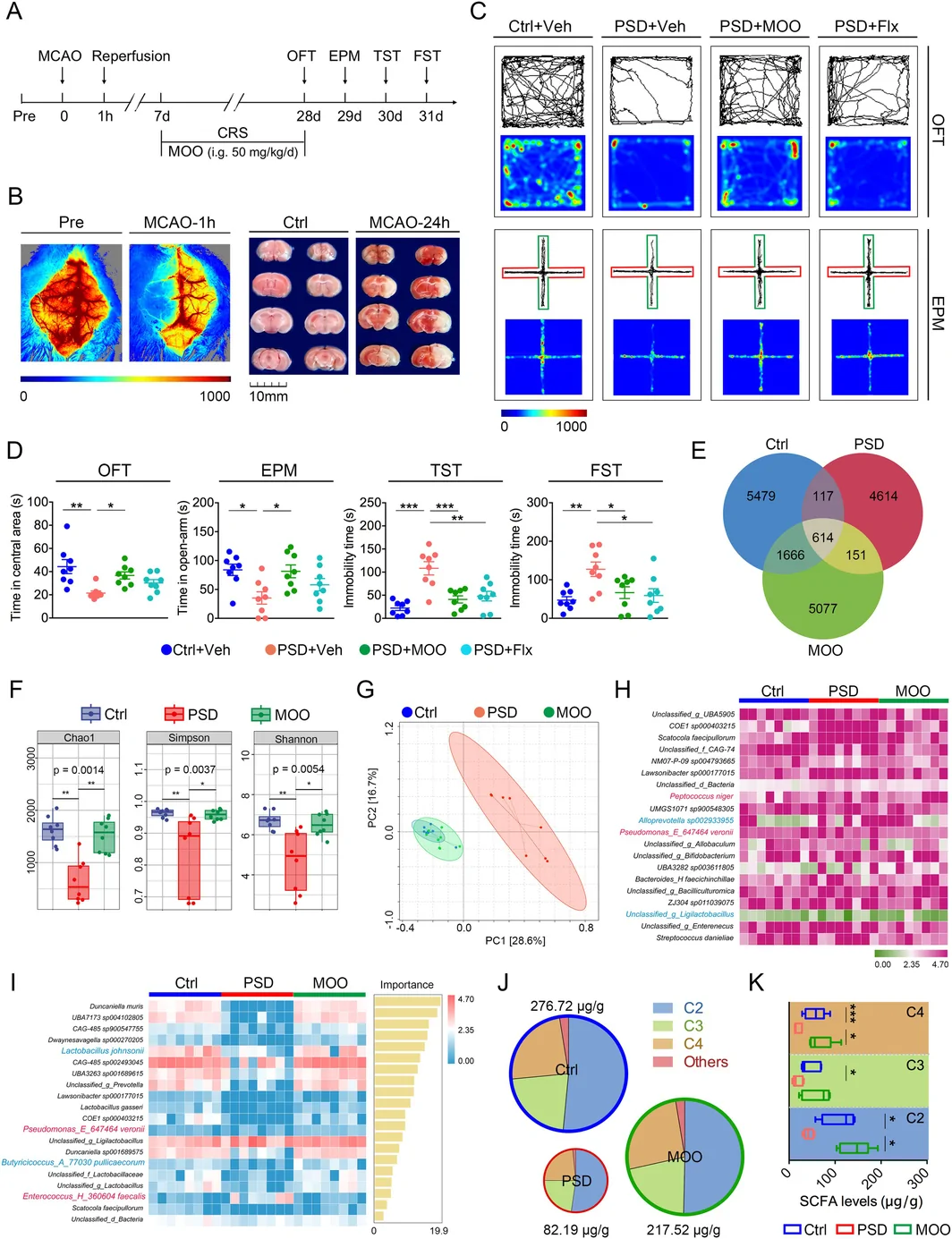
MOO ameliorates depressive‐like behaviors in PSD mice and partially restores gut microbial homeostasis. (A) Experimental design. CRS, chronic restraint stress; EPM, elevated plus maze; FST, forced swim test; MCAO, middle cerebral artery occlusion; MOO, morinda officinalis oligosaccharides; OFT, open field test; TST, tail suspension test. (B) Representative images of cerebral blood flow (CBF) by laser speckle contrast imaging (LSCI); representative images of TTC‐stained brain slices from an independent model‐validation cohort that was not used for subsequent behavioral or molecular experiments. Scale bars: 10 mm. (C) Representative movement tracks and heat maps in the OFT and EPM; Ctrl: Control; Flx, fluoxetine; MOO, morinda officinalis oligosaccharides; PSD, post‐stroke depression; Veh, vehicle. (D) Time spent in the central area of the OFT; duration in the open arms of the EPM; immobility time in the TST and FST. Significance was determined by one‐way ANOVA with Tukey's post hoc test, *n* = 8, *p < 0.05, **p < 0.01, ***p < 0.001. (E) Venn diagram showing shared amplicon sequence variants (ASVs) among the Ctrl, PSD, and MOO groups. (F) Alpha diversity of the gut microbiota among the Ctrl, PSD, and MOO groups, assessed using the Chao1, Simpson, and Shannon indices. Overall differences among groups were evaluated using the Kruskal–Wallis test, followed by Dunn's multiple‐comparisons test for post hoc pairwise comparisons, *n* = 8, *p < 0.05, **p < 0.01. (G) Beta diversity of the gut microbiota was assessed by principal coordinate analysis (PCoA) based on Bray–Curtis dissimilarity, *n* = 8. (H) Heatmap of discriminatory bacterial taxa identified using a support vector machine (SVM), showing their abundance patterns across the Ctrl, PSD, and MOO groups, *n* = 8. (I) Heatmap of bacterial taxa selected using a random forest (RF) model. The bar plot on the right indicates the relative importance of each taxon in the random forest model, *n* = 8. (J) Composition characteristics: Pie charts show the relative abundance and mass concentration of short‐chain fatty acids (SCFAs) in each group. C2, Acetate; C3, Propionate; C4, Butyrate. (K) Quantitative comparison: Box‐and‐whisker plots comparing fecal acetate (C2), propionate (C3), and butyrate (C4) concentrations. Statistical significance was determined by one‐way ANOVA followed by Tukey's post hoc test; *n* = 5 per group. *p < 0.05, ***p < 0.001.

To assess the potential direct impact of intragastric MOO on the gut environment [19], we profiled concomitant changes in the gut microbiota via 16S rRNA sequencing. A Venn diagram identified a core of 614 common amplicon sequence variants (ASVs) shared across the Ctrl, PSD, and MOO groups (Figure 1E). Alpha diversity analysis showed that MOO intervention significantly increased the Chao1 index, Simpson index, and Shannon index compared to the PSD group (Figure 1F). Additional alpha diversity metrics, including Goods coverage, Pielou's evenness, Faith's phylogenetic diversity (Faith_pd), and Observed species, further confirmed that MOO treatment enhanced microbial richness and evenness compared with the PSD group (Figure S1E). Beta diversity visualization showed separation between the PSD and Ctrl samples, whereas MOO samples clustered closer to and partially overlapped with Ctrl samples, suggesting that MOO treatment was associated with changes in gut microbial community structure in PSD mice (Figure 1G). Taxonomic composition analysis at the family and genus levels was further visualized by stacked bar plots of relative abundance (Figure S1D), which illustrated the compositional differences among the three groups. The results showed that the gut microbial structure of PSD mice was markedly altered compared with the Ctrl group, while MOO intervention partially reversed the PSD‐induced dysbiosis.

To identify microbial features that could distinguish among the three groups, we used a support vector machine (SVM) algorithm to select 19 selected microbial taxa and generated a heatmap of their relative abundances (Figure 1H). We then applied a random forest model to evaluate the contribution of each species to group discrimination and generated a heatmap of the selected microbial features (Figure 1I). The results showed that beneficial taxa identified by the random forest model were more abundant in the Ctrl group, decreased in the PSD group, and partially restored following MOO intervention. In contrast, opportunistic pathogens tended to be enriched in the PSD group, and MOO intervention effectively reduced their relative abundances.

To assess the transferability of fecal effects associated with MOO treatment, we used four PSD‐background groups: PSD + Veh, PSD + MOO, FMT‐PSD, and FMT‐MOO (Figure S1F–H). Compared with FMT‐PSD recipients, FMT‐MOO recipients spent significantly more time in the OFT central zone and EPM open arms and showed significantly less immobility in the TST and FST (Figure S1G). The PSD‐versus‐MOO direct‐treatment comparison also showed significant improvements in all four measures. Representative OFT and EPM tracks and heatmaps are shown in Figure S1H. These findings provide preliminary evidence that fecally transferable factors from MOO‐treated PSD donors may contribute to improvements in depression‐like behaviors in PSD recipient mice compared with those receiving fecal material from vehicle‐treated PSD donors.

SCFAs, primarily including acetate (C2), propionate (C3), and butyrate (C4), are important metabolites derived from the gut microbiota [20]. Analysis of SCFAs revealed markedly reduced total levels in the PSD group (82.19 μg/g) compared with the Ctrl group (276.72 μg/g), particularly for C4. MOO intervention significantly restored the total SCFA content (217.52 μg/g) and shifted the composition toward control levels (Figure 1J,K). These findings show that PSD and MOO treatment were associated with differences in fecal SCFA composition and levels.

### 3.2 MOO Treatment Is Associated With Improved Intestinal and Blood–Brain Barrier in Post‐Stroke Depression

To investigate the protective effects of MOO on the gut‐brain axis, we examined key intestinal barrier components. Colonic IF analysis revealed that PSD significantly reduced E‐cadherin expression, a critical structure for intercellular adhesion and barrier integrity [21]. This loss was robustly reversed by MOO treatment (Figure 2A,B). To further characterize the intestinal barrier damage, we examined three major intestinal tight junction proteins (Claudin‐1, ZO‐1, and Occludin) by IF and WB. Both analyses consistently revealed significant downregulation of these proteins in the PSD group, which was partially reversed following MOO treatment (Figure 2C,D).

**FIGURE 2:**
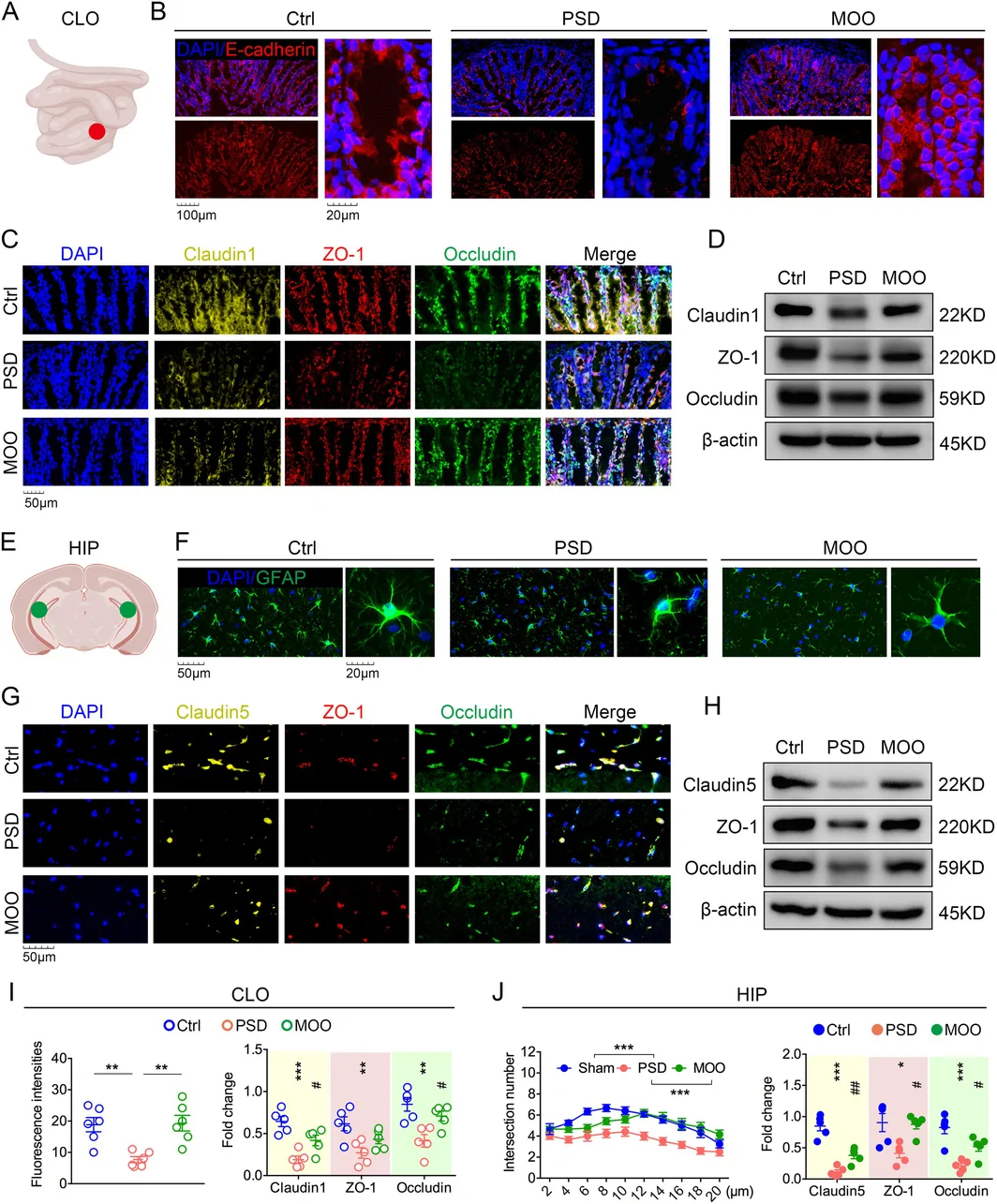
MOO treatment is associated with improved intestinal and blood–brain barrier in post‐stroke depression. (A) Diagram of colonic (CLO) location. (B) Representative immunofluorescence staining images of E‐cadherin (red) co‐labeled with DAPI (blue) in the colon. Ctrl, control; PSD, post‐stroke depression; MOO, morinda officinalis oligosaccharides. (C) Representative immunofluorescence staining images of Claudin‐1 (yellow), ZO‐1 (red), and Occludin (green) in the colon. (D) Representative western blotting (WB) images of Claudin‐1, ZO‐1, and Occludin in the colonic tissues across the three groups. (E) Diagram of hippocampus (HIP) location; green circles indicate selected hippocampal regions used for immunofluorescence quantification. (F) Representative immunofluorescence staining images of GFAP (green) co‐labeled with DAPI (blue) in the hippocampus. (G) Representative immunofluorescence staining images of Claudin‐5 (yellow), ZO‐1 (red), and Occludin (green) in the hippocampus. (H) Representative western blotting images of Claudin‐5, ZO‐1, and Occludin in hippocampal tissues across the three groups. (I) Representative quantification of E‐cadherin fluorescence intensity in the colon and fold‐change analysis of Claudin‐1, ZO‐1, and Occludin protein levels in the colon by Western blot (WB). For fluorescence intensities, significance was determined by one‐way ANOVA with Tukey's post hoc test, *n* = 6, **p < 0.01. For WB fold change, significance was determined by one‐way ANOVA with Tukey's post hoc test, *n* = 5, **p < 0.01, ***p < 0.001, the PSD group versus the Ctrl group; ^#^ *p* < 0.05, the MOO group versus the PSD group. (J) Sholl analysis of astrocyte morphology in the hippocampus, showing the number of intersections as a function of distance from the cell center, and fold‐change analysis of Claudin‐5, ZO‐1, and Occludin protein levels in the hippocampus by Western blot (WB). For Sholl analysis, significance was determined by two‐way ANOVA with Tukey's post hoc test, *n* = 20 from 5 mice, ***p < 0.001. For WB fold change, significance was determined by one‐way ANOVA with Tukey's post hoc test, *n* = 5, *p < 0.05, ***p < 0.001, the PSD group versus the Ctrl group; ^#^ *p* < 0.05, ^##^ *p* < 0.01, the MOO group versus the PSD group. Data are presented as the mean ± SEM.

Given the observed impairment of the intestinal barrier, we next examined whether gut‐derived inflammatory signals might affect the BBB. Indeed, the Evans blue assay confirmed BBB impairment in PSD mice, while ELISA revealed a significant increase in inflammatory cytokine levels in the hippocampus (Figure S2A–C). Moreover, hippocampal IF revealed striking atrophic changes in GFAP^+^ astrocytes, key constituents of the BBB, in PSD mice, characterized by somal shrinkage and process retraction. MOO treatment largely reversed these morphological deficits (Figure 2E,F). Parallel impairment was observed in the expression of the hippocampal tight junction proteins (Claudin‐5, ZO‐1, Occludin). Consistent with the intestinal findings, their expression was significantly suppressed in PSD mice and rescued by MOO intervention (Figure 2G,H). Quantitative analyses of the above IF and WB results confirmed that MOO treatment significantly restored the expression of intestinal tight junction proteins (Claudin‐1, ZO‐1, and Occludin) and E‐cadherin, as well as hippocampal tight junction proteins (Claudin‐5, ZO‐1, and Occludin), and reversed the morphological atrophy of GFAP^+^ astrocytes (Figure 2I,J).

To further assess whether the restoration of barrier integrity by MOO was associated with functional recovery of neuronal activity in the brain, we examined c‐Fos expression in the hippocampus. IF staining revealed that PSD significantly reduced the number of c‐Fos^+^ cells in the hippocampal region, indicating suppressed neuronal activity. MOO treatment significantly reversed this reduction, suggesting enhanced neuronal activation (Figure S2D,E). Double immunofluorescence staining further revealed that the majority of c‐Fos^+^ cells co‐localized with the glutamatergic neuronal marker CaMKIIα, indicating that the MOO‐activated neurons were primarily glutamatergic neurons (Figure S2F).

Collectively, MOO treatment was associated with improved indices of intestinal epithelial and hippocampal BBB integrity and with increased hippocampal neuronal activity in PSD mice.

### 3.3 Increased Pla2g4a Expression Underlies PSD‐Associated Lipid Metabolism and Signal Transduction Pathways

To explore hippocampal molecular changes associated with the protective effects of MOO, we undertook a comprehensive hippocampal proteomic analysis (Figure 3A). Heatmaps of the differentially expressed proteins (DEPs) illustrated the overall expression profiles between the PSD and Ctrl groups, as well as between the MOO and PSD groups, with specific protein names annotated (Figure S3A,B). The analysis identified 55 upregulated and 40 downregulated proteins in the PSD group compared to the Ctrl group (Figure 3B) and 49 upregulated and 47 downregulated proteins in the MOO group compared to the PSD group (Figure 3C). Subcellular localization analysis showed that these DEPs were predominantly distributed in the cell membrane and cytoplasm (Figure S3C,D). Subsequent Gene Ontology (GO) enrichment analysis of the differentially expressed proteins (DEPs) identified significant terms including “Cellular component organization or biogenesis,” “Cellular anatomical entity,” and “Protein binding” (Figure 3D,E). Concurrently, Kyoto Encyclopedia of Genes and Genomes (KEGG) pathway analysis revealed their prominent involvement in “Alpha‐linolenic acid metabolism,” “Calcium signaling pathway,” and “cAMP signaling pathway” (Figure 3F,G).

**FIGURE 3:**
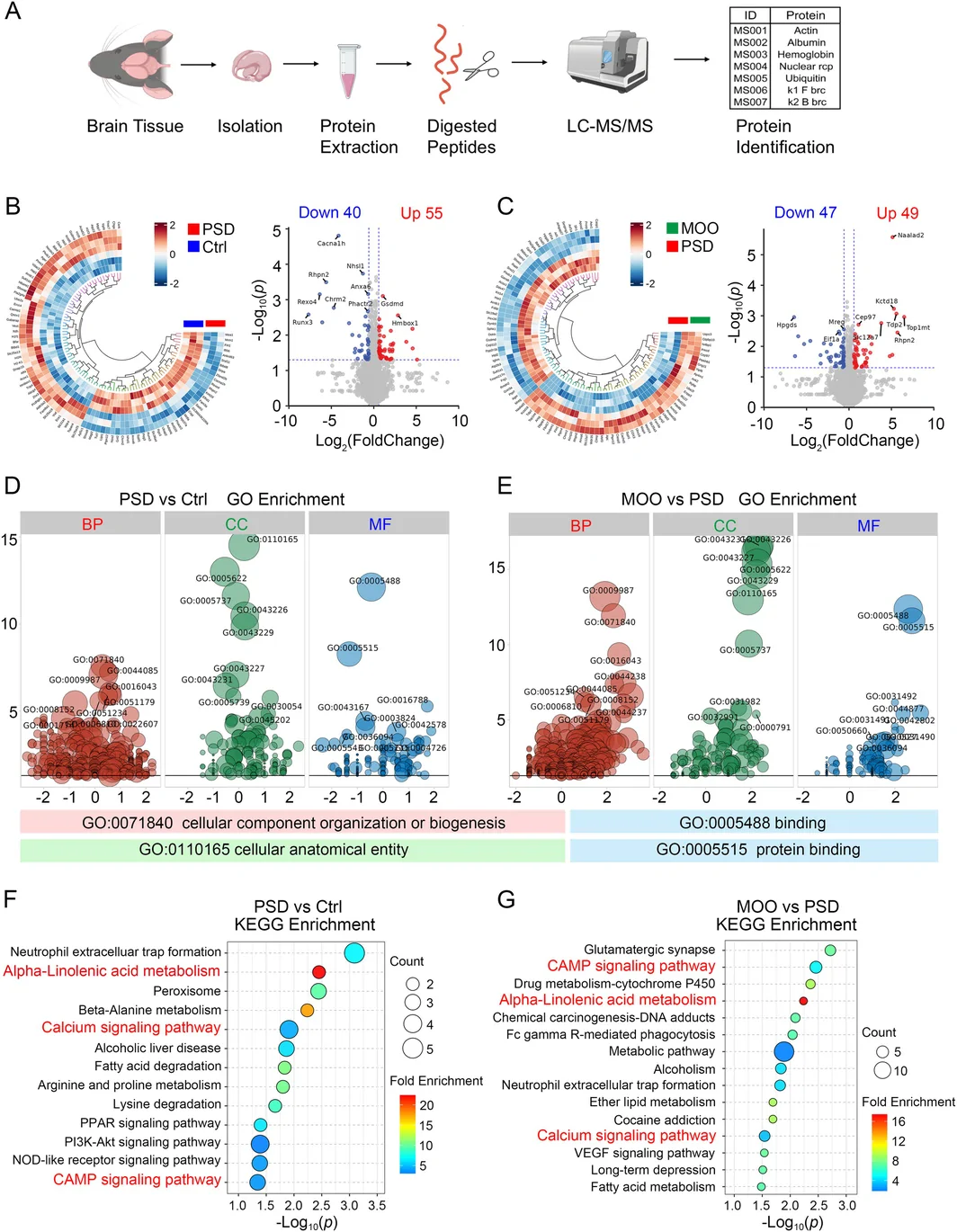
Proteomic profiling of hippocampal DEPs and pathway enrichment in PSD mice following MOO treatment. (A) Schematic diagram of proteomic analysis, *n* = 3. (B, C) Volcano plot and heatmap of differentially expressed proteins (labeled by gene names). (D–G) Gene Ontology (GO) and Kyoto Encyclopedia of Genes and Genomes (KEGG) pathway enrichment analyses of differentially expressed proteins. Ctrl, control; PSD, post‐stroke depression; MOO, morinda officinalis oligosaccharides.

A clustering heatmap of the DEPs revealed the broad regulatory effect of MOO on the hippocampal proteome (Figure 4A), while a subsequent Venn analysis identified a core set of 9 DEPs whose PSD‐induced upregulation was reversed by MOO intervention (Figures 4B and S4A). Further KEGG analysis revealed that the overlapping DEPs converged predominantly on “Lipid metabolism” and “Signal transduction” pathways. This core set included several hub proteins: Acox1, a key enzyme in fatty acid β‐oxidation; cytosolic phospholipase A2α (Pla2g4a); and the neurotrophic factor brain‐derived neurotrophic factor (BDNF) (Figures 4C and S4B,C). In addition, we examined the opposite direction of regulation: proteins that were downregulated by PSD and subsequently upregulated by MOO treatment (Figure S4D–F).

**FIGURE 4:**
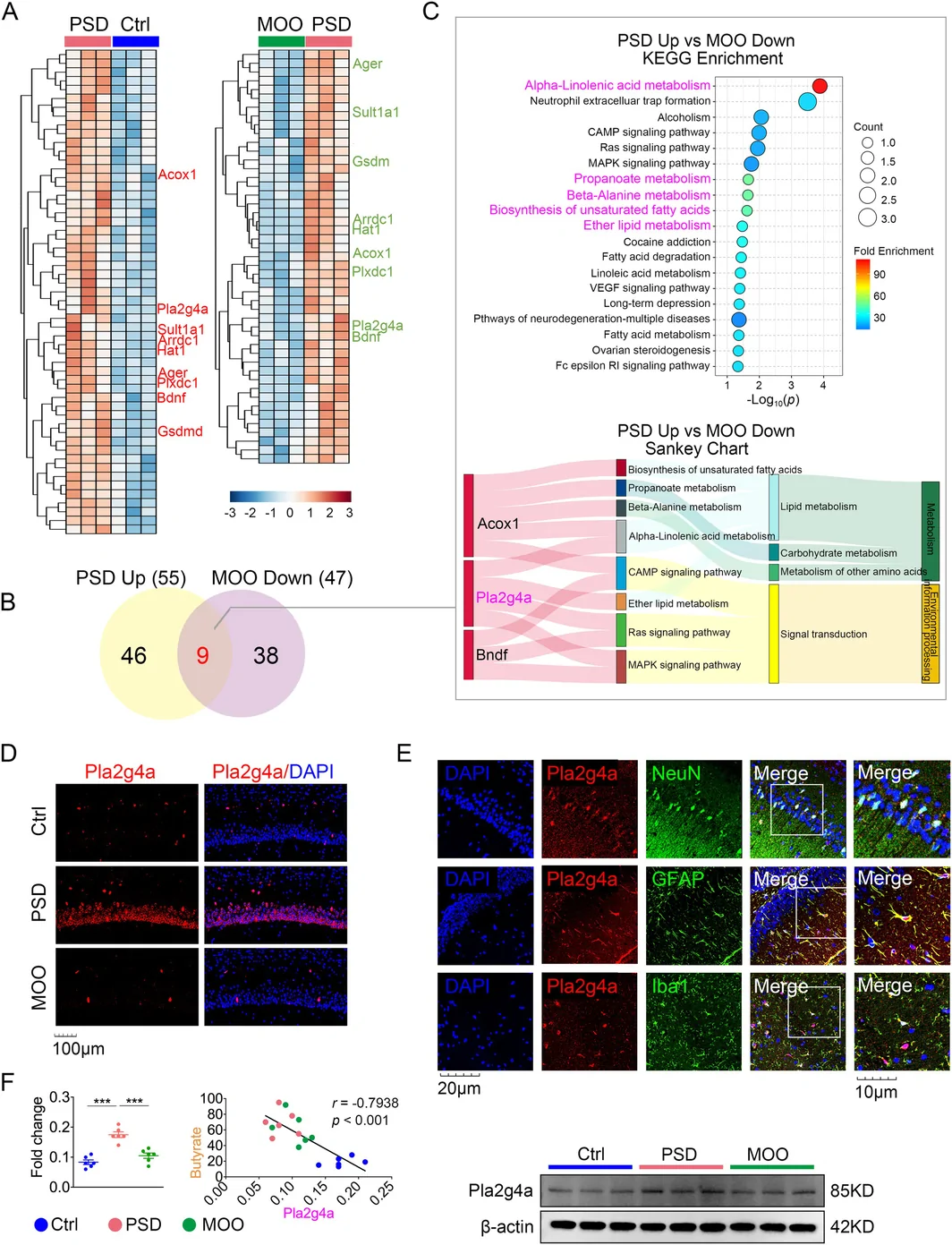
Increased Pla2g4a expression underlies PSD‐associated lipid metabolism and signal transduction pathways. (A) Heatmap of differentially expressed proteins (labeled by gene names). (B) Venn diagram depicting the overlap between the DEPs that were upregulated in the PSD group and downregulated in the MOO group. (C) Sankey chart of Kyoto Encyclopedia of Genes and Genomes (KEGG) pathway enrichment analysis of 9 overlapping DEPs. (D) Representative immunostaining images of Pla2g4a (red) co‐labeled with DAPI (blue) in the hippocampus. (E) Representative immunofluorescence images showing colocalization of Pla2g4a (red) with the neuronal marker NeuN (green), the astrocyte marker GFAP (green), and the microglial marker Iba1 (green) in the hippocampus of the PSD group. Scale bars = 20 or 10 μm. (F) Fold change of Pla2g4a, correlation analysis between butyrate and Pla2g4a, and representative western blot (WB) images of Pla2g4a (with β‐actin as a loading control) in Ctrl, PSD, and MOO groups. For WB fold change, significance was determined by one‐way ANOVA with Tukey's post hoc test, *n* = 6, ***p < 0.001. For correlation analysis, *n* = 6, *r* = −0.7938, *p* < 0.001. Data are presented as the mean ± SEM. Ctrl, control; PSD, post‐stroke depression; MOO, morinda officinalis oligosaccharides.

Among these candidates, Pla2g4a encodes cytosolic phospholipase A2α (cPLA2α), a rate‐limiting enzyme that catalyzes the hydrolysis of membrane phospholipids to release arachidonic acid, positioning it as an upstream regulator of neuroinflammatory cascades implicated in mood disorders [22]. IF and WB analyses validated the proteomic findings, confirming that Pla2g4a expression was higher in the PSD group and lower following MOO treatment. We further examined the cellular localization of Pla2g4a in the hippocampus. In the PSD group, Pla2g4a immunoreactivity was predominantly detected in neurons but was also observed in Iba1‐positive microglia and GFAP‐positive astrocytes, indicating contributions from multiple cell types (Figure 4E). C4 levels and Pla2g4a expression were inversely correlated (Figure 4D,F). This association, together with the intervention data, identifies Pla2g4a as a candidate molecule for further mechanistic investigation.

### 3.4 Butyrate Alleviates Behavioral, Intestinal Barrier, and Blood–Brain Barrier Deficits While Suppressing Pla2g4a

To further investigate the potential role of butyrate (C4) in PSD, sodium butyrate (SB; 30 mg/kg/day) was administered once daily by oral gavage from Day 7 to Day 27 during the PSD modeling period. Behavioral assessments were conducted to evaluate treatment efficacy (Figure 5A). Pie charts depict the total amounts and compositional proportions of SCFAs (including C2, C3, C4, and others) across the Ctrl, PSD, and SB groups. The PSD group exhibited significantly reduced total SCFA levels, whereas SB supplementation increased total SCFA levels and partially shifted the SCFA composition toward that of the Ctrl group (Figure 5B). The increase in fecal C4 content in the SB group further confirmed the successful elevation of butyrate availability following exogenous supplementation. Notably, SB treatment was also associated with significantly increased butyrate concentrations in serum and hippocampal tissue (Figure 5C), indicating increased systemic and hippocampal exposure to butyrate. Compared with Ctrl mice, PSD mice spent significantly less time in the central zone of the OFT and the open arms of the EPM. SB treatment significantly increased the time spent in the OFT central zone compared with the PSD group, while the time spent in the EPM open arms showed an increasing tendency without reaching statistical significance. In the TST and FST, PSD mice exhibited significantly increased immobility, both of which were significantly reduced by SB treatment (Figure 5D,E). Collectively, these findings indicate that SB treatment alleviates depressive‐like behavioral abnormalities in PSD mice, with significant improvements observed in the OFT, TST, and FST.

**FIGURE 5:**
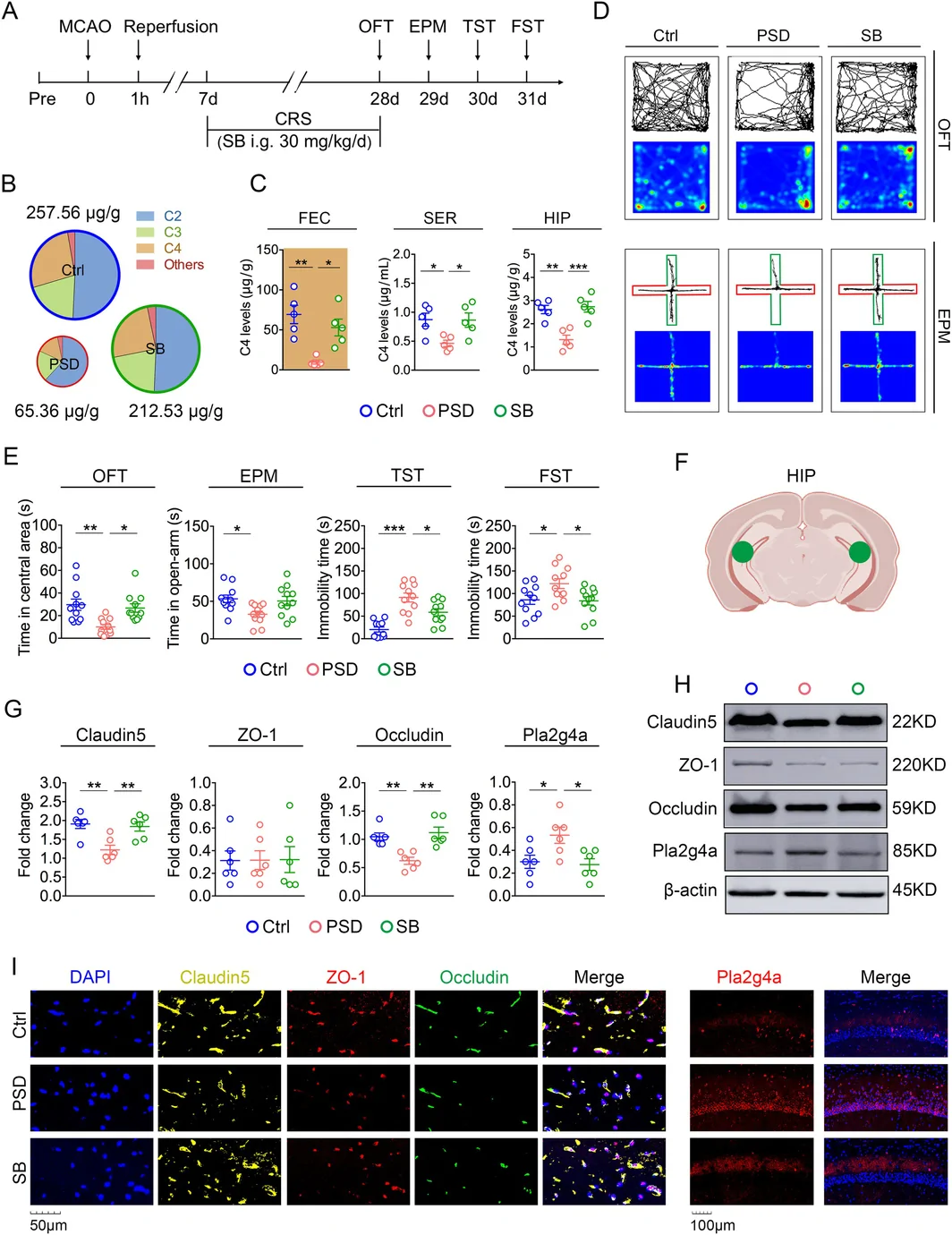
Butyrate alleviates behavioral, intestinal barrier, and blood–brain barrier deficits while suppressing Pla2g4a. (A) Experimental design. MCAO, middle cerebral artery occlusion; OFT, open field test; EPM, elevated plus maze; TST, tail suspension test; FST, forced swim test; CRS, chronic restraint stress; SB, sodium butyrate. (B) Composition characteristics: Pie charts show the relative abundance and mass concentration of short‐chain fatty acids (SCFAs) in each group. Ctrl, control; PSD, post‐stroke depression; SB, sodium butyrate; C2, acetate; C3, propionate; C4, butyrate. (C) Quantification of C4 (butyrate) levels in fecal (μg/g), serum (μg/mL), and hippocampal (μg/g). A scatter plot with the mean ± SEM illustrates the levels. Statistical comparisons were performed by one‐way ANOVA with Tukey's post hoc test, *n* = 5, *p < 0.05, **p < 0.01, ***p < 0.001. (D) Representative movement tracks and heat maps in the OFT and EPM. (E) Time spent in the central area of the OFT; duration in the open arms of the EPM; immobility time in the TST and FST. Significance was determined by one‐way ANOVA with Tukey's post hoc test, *n* = 11, *p < 0.05, **p < 0.01, ***p < 0.001. (F) Diagram of hippocampus (HIP) location; green circles indicate selected hippocampal regions used for immunofluorescence quantification. (G, H) Quantification and representative western blotting images of Claudin‐5, ZO‐1, Occludin and Pla2g4a (with β‐actin as a loading control). Significance was determined by one‐way ANOVA with Tukey's post hoc test, *n* = 6, *p < 0.05, **p < 0.01. Data are presented as the mean ± SEM. (I) Representative immunofluorescence images of Claudin‐5, ZO‐1, Occludin, and Pla2g4a. Merged images with DAPI staining show protein expression in the Ctrl, PSD, and SB groups.

We next investigated whether SB treatment was associated with improvements in PSD‐related intestinal and BBB dysfunction. Quantitative IF and WB analyses showed that SB treatment partially restored the expression of barrier‐associated tight junction proteins in both the intestine and hippocampus. It partially reversed the PSD‐induced downregulation of Claudin‐1, ZO‐1, and Occludin in the colon (Figure S5) and similarly restored hippocampal Claudin‐5, ZO‐1, and Occludin expression (Figure 5G–I). The protein level of Pla2g4a, which was significantly elevated in the model group, was markedly reduced by SB intervention (Figure 5H,I). This suppression aligns with their noted negative correlation, suggesting that exogenous SB may exert its therapeutic effects, at least in part, through modulation of Pla2g4a‐associated pathways in PSD.

### 3.5 Hippocampal Pla2g4a Overexpression Attenuates Sodium Butyrate‐Induced Behavioral Improvement in PSD Mice

Finally, to investigate the functional role of Pla2g4a in the response to sodium butyrate, we overexpressed Pla2g4a in the hippocampus using a CMV promoter‐driven adeno‐associated virus (AAV). Pla2g4a overexpression was first verified by Western blotting in plasmid‐transfected 293T cells, which showed significantly higher Pla2g4a protein levels than the negative control (NC) group (Figure 6A). The AAV vector was then stereotaxically injected into the bilateral hippocampi of mice. Western blotting of hippocampal tissues collected 21 days after AAV injection confirmed significantly increased Pla2g4a protein levels compared with the NC group (Figure 6B), and immunofluorescence staining verified successful transduction of the target regions (Figure 6C). Behavioral tests confirmed robust depressive‐like behaviors in PSD mice (PSD + NC + Veh), as evidenced by reduced exploration in the OFT center and EPM open arms, and prolonged immobility in TST/FST. SB treatment (PSD + NC + SB) significantly ameliorated these behavioral deficits. Crucially, hippocampal Pla2g4a overexpression (PSD + Pla2g4a + SB) partially attenuated the therapeutic benefit of SB across all tests (Figure 6D,E). These findings suggest that hippocampal Pla2g4a is functionally involved in the behavioral response to sodium butyrate treatment and may represent a candidate downstream mediator of its antidepressant‐like effects.

**FIGURE 6:**
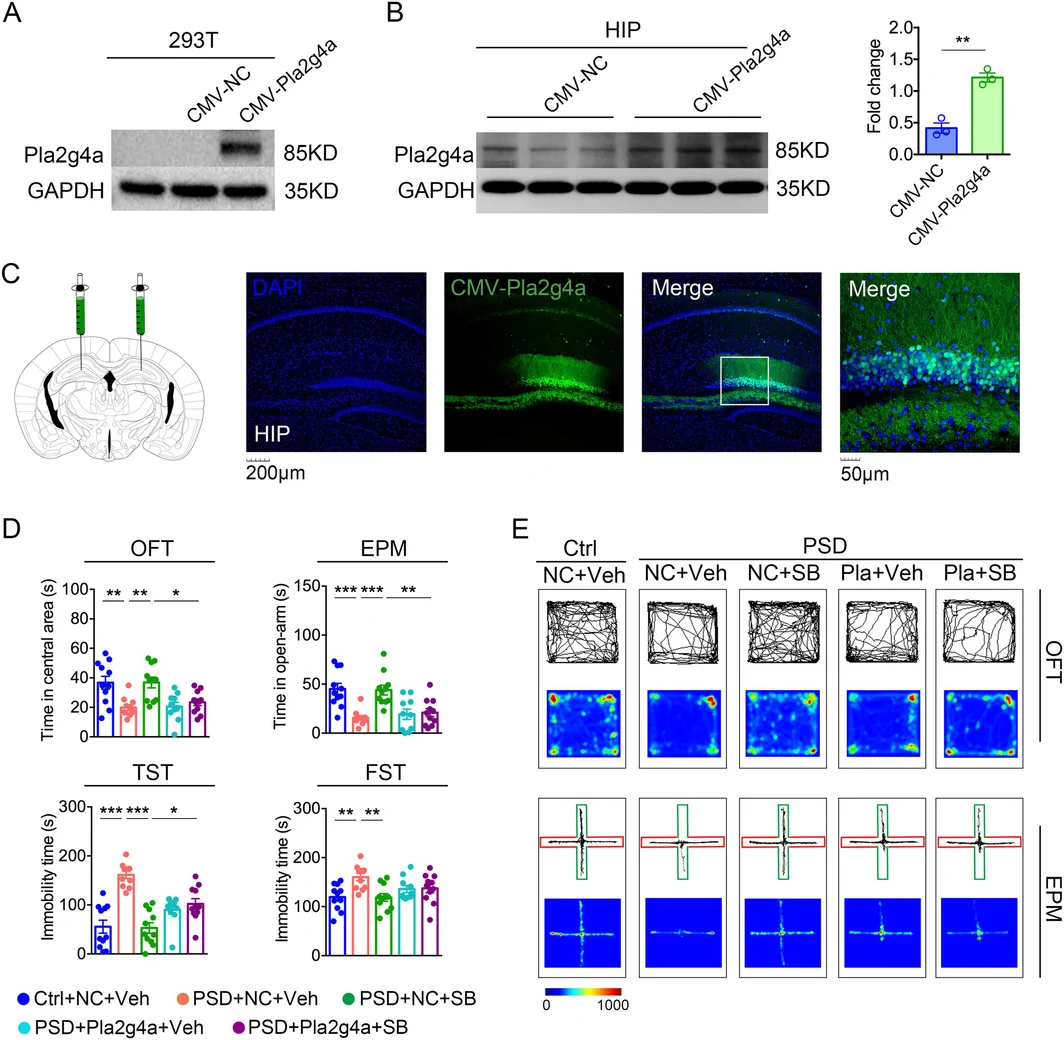
Hippocampal Pla2g4a overexpression attenuates sodium butyrate‐associated behavioral improvement in PSD mice. (A) Representative western blot of Pla2g4a protein levels in 293T cells following transfection with CMV‐NC or CMV‐Pla2g4a plasmids, using GAPDH as a loading control. (B) Representative Western blot and quantitative analysis of Pla2g4a protein levels in hippocampal tissues collected 21 days after AAV injection. Significance was determined by unpaired t‐test, *n* = 3, **p < 0.01. Data are presented as the mean ± SEM. (C) Representative immunofluorescence staining of CMV‐Pla2g4a (green) co‐labeled with DAPI (blue) in the hippocampus, with the merged image demonstrating cellular localization. (D) Time spent in the central area of the OFT; duration in the open arms of the EPM; immobility time in the TST and FST. Significance was determined by one‐way ANOVA with Tukey's post hoc test, *n* = 11, *p < 0.05, **p < 0.01, ***p < 0.001. Data are presented as the mean ± SEM. (E) Representative movement tracks and heat maps in the OFT and EPM; Ctrl, control; Pla, Pla2g4a; PSD, post‐stroke depression; SB, sodium butyrate; Veh, vehicle.

## 4 Discussion

MOO alleviated gut microbiota dysbiosis in PSD mice, as evidenced by the restoration of α‐ and β‐diversity, an increased abundance of beneficial bacteria, and a decreased abundance of potentially harmful bacteria. This finding is consistent with observations in a rat model of depression, in which MOO can reverse the abnormal increase of depression‐related bacteria such as *Anaerostipes*, *Oscillibacter*, and *Streptococcus*, suggesting that MOO can exert antidepressant effects by remodeling intestinal microflora [23]. This was accompanied by elevated total SCFAs, especially butyrate (Figure 1). Moreover, MOO promoted the repair of both intestinal and hippocampal barriers, partially restored expression of intestinal tight junction proteins (Claudin‐1, ZO‐1, Occludin) and hippocampal tight junction proteins (Claudin‐5, ZO‐1, Occludin), while also mitigating astrocyte atrophy (Figure 2).

The potential mechanisms underlying these protective effects may involve changes in SCFA levels. SCFAs serve as primary energy sources for intestinal epithelial cells, and butyrate has been reported to reinforce intestinal physical barrier integrity by activating G‐protein‐coupled receptors (e.g., GPR43/109A) and inhibiting histone deacetylase (HDAC), thereby promoting tight junction assembly via dual pathways [24]. Additionally, the microbiota associated with MOO treatment may modulate local gut and systemic immune homeostasis through SCFAs and bacterial components (e.g., polysaccharide A), consequently reducing systemic inflammation [25], consistent with our observations. Such immunomodulation may contribute to BBB maintenance because systemic inflammation accompanying PSD can promote BBB disruption and neuroinflammation. A pharmacokinetic study of MOO showed that the peak blood concentration of FOS‐DP5, a representative MOO component, was approximately 900 ng/mL after oral administration and decreased below the detection limit after 4 h. Systemic exposure was much lower than after intravenous administration, supporting low intestinal permeability and limited systemic entry after oral dosing [11]. Together, these findings support further investigation of gut‐associated mechanisms without establishing that intestinal microenvironment repair is required for the neurobehavioral effects of MOO.

Through hippocampal proteomic analysis, we identified Pla2g4a as a candidate molecule within the MOO‐mediated pathway (Figures 3 and 4). Pla2g4a encodes cytosolic phospholipase A2α (cPLA2α), a rate‐limiting enzyme that hydrolyzes the sn‐2 ester bond of membrane phospholipids in neurons and glial cells upon activation by upstream signals such as pro‐inflammatory cytokines and reactive oxygen species, releasing arachidonic acid (AA). The released AA serves not only as the direct substrate for synthesizing classic pro‐inflammatory mediators like prostaglandins (PGs) and leukotrienes (LTs) but also functions as a lipid second messenger, amplifying inflammatory signaling cascades [26]. In stress‐induced depression models, hippocampal Pla2g4a expression has been reported to be significantly elevated, with its activity correlating positively with pro‐inflammatory cytokine levels and the severity of depressive‐like behaviors [27]. These observations suggest that Pla2g4a may contribute to neuroinflammatory processes relevant to PSD. Importantly, an independent investigation using TMT‐based quantitative proteomics combined with network pharmacology identified Pla2g4a as a candidate differential protein in the MCAO rat model of ischemic stroke [28]. In that study, targeted inhibition of Pla2g4a in an in vitro PC12 cell ischemia model significantly reduced arachidonic acid and COX‐2 release and promoted calcium influx, thereby exerting clear neuroprotective effects. This independent evidence from the ischemic stroke setting is highly consistent with our proteomic findings and corroborates the potential role of Pla2g4a in ischemic stroke pathology. Given the well‐established link between ischemic injury and post‐stroke depression, these converging lines of evidence further support our conclusion that Pla2g4a is involved in the butyrate response pathway and may serve as a candidate downstream mediator of SB's antidepressant‐like effects.

Beyond its role in neuroinflammation, Pla2g4a overactivation has been linked to abnormal cell membrane phospholipid composition, altered membrane fluidity, and disrupted lipid raft structure [29]. Recent research highlights that the neuronal membrane lipid microenvironment may contribute to the localization and signal transduction of the BDNF receptor, TrkB. Pla2g4a‐mediated lipid hydrolysis disrupts TrkB enrichment within lipid rafts, thereby blunting BDNF signaling and potentially impairing neuroplasticity and cognitive function [22]. Furthermore, Pla2g4a has been reported to induce lysosomal membrane permeabilization and inhibit autophagic flux, leading to the accumulation of toxic proteins within neurons, a mechanism validated in traumatic brain injury and Alzheimer's disease [30]. These insights provide a novel perspective for understanding the potential role of Pla2g4a in PSD.

Multiple large‐scale clinical studies have confirmed reduced blood butyrate levels in patients with major depressive disorder (MDD), showing a negative correlation with Hamilton Depression Rating Scale (HAMD‐17) scores [31]. In this study, exogenous SB supplementation in PSD model mice improved depressive‐like behaviors, as evidenced by increased exploration time in the OFT and EPM, and decreased immobility time in the TST and FST (Figure 5). This aligns with the research indicating that SB exhibits antidepressant effects in the Chronic Unpredictable Mild Stress (CUMS) model, not only improving anhedonia and behavioral despair but also repairing hippocampal synaptic plasticity impairment via epigenetic mechanisms [32]. Additionally, specific probiotics like *Akkermansia muciniphila* can ameliorate depressive‐like behaviors by elevating gut butyrate levels [33]. At the mechanistic level, an in vitro single‐strain culture study suggested that MOO could increase the level of 5‐HTP and decrease the level of 5‐HT in the *Clostridium butyricum* culture system, indicating that this strain may be one of the important candidates for mediating MOO‐regulated intestinal 5‐HTP/5‐HT metabolism [10]. This provides direct strain‐level evidence that MOO can affect neurotransmitter precursor metabolism through specific gut microbiota. These findings provide support for the potential role of butyrate in depression and underscore its potential as a messenger within the MGBA.

As a primary metabolite of colonic microbial fermentation of dietary fiber, butyrate has been reported to strengthen the BBB through multiple mechanisms: it may act on cerebrovascular endothelial cells to promote tight junction protein expression; it may enhance neurovascular unit stability by regulating pericyte function [34]; and it may ameliorate stress‐induced BBB leakage by modulating microglia‐endothelial cell interactions [35]. In our study, SB treatment was associated with higher expression of hippocampal tight junction proteins (Claudin‐5, ZO‐1, and Occludin) in PSD mice. LC–MS/MS analysis showed higher butyrate levels in serum and hippocampal after SB treatment. SB treatment was associated with reduced hippocampal Pla2g4a protein levels (Figure 5), and butyrate levels were inversely correlated with Pla2g4a expression. These findings support an association between SB treatment and Pla2g4a‐related changes; direct molecular regulation remains to be tested using mechanistic experiments.

Based on these findings, we propose a working model in which oral MOO is associated with changes in gut microbial composition and fecal SCFA levels, including butyrate. MOO and sodium butyrate treatment were also associated with improved barrier‐related measures and lower hippocampal Pla2g4a expression. Whether these observations form a direct causal sequence, and whether circulating butyrate enters the brain parenchyma to regulate Pla2g4a remain unresolved.

To investigate the functional involvement of Pla2g4a in the butyrate response pathway, hippocampal overexpression of Pla2g4a was performed. Pla2g4a overexpression partially attenuated the antidepressant effects of butyrate, reducing exploratory activity and increasing immobility in behavioral tests (Figure 6). This finding supports the involvement of Pla2g4a as a candidate downstream mediator linking gut metabolites to central behavioral regulation, and is consistent with the emerging concept that “microbial metabolites may influence neuropsychiatric disorders by modulating brain lipid metabolism” [36].

Several limitations of this study should be acknowledged. First, only male mice were used, and the findings may not generalize to females. Second, the PSD model combines MCAO with chronic restraint stress; whether the Pla2g4a findings hold in other PSD paradigms remains unknown. Third, although donor‐controlled FMT supports the transferability of MOO‐associated fecal effects, residual MOO, metabolites, and other soluble fecal components were not quantified; sterile‐filtrate, heat‐killed, and microbiota‐depletion controls are needed to identify the active transferable component and determine whether viable microbiota is required [37]. Fourth, although LC–MS/MS quantified butyrate in hippocampal, serum, and fecal samples, these measurements cannot distinguish whether butyrate crosses the blood–brain barrier and enters hippocampal parenchyma. Future studies are needed to determine whether butyrate enters the hippocampus and how it influences Pla2g4a expression. Fifth, TTC staining was performed in an independent terminal cohort; baseline infarct volumes were not measured in the same animals used for behavioral and molecular experiments, so equivalence of initial infarct severity among treatment groups cannot be confirmed. Finally, no human data are provided; therefore, Pla2g4a should be considered a candidate molecule rather than an established therapeutic target for PSD.

The integrated findings identify coordinated associations among MOO treatment, the gut microenvironment, barrier‐related measures, and hippocampal Pla2g4a expression. Pla2g4a may be a candidate molecule linking peripheral metabolic changes to brain function, but the direction and molecular basis of these relationships require further causal validation.

## Author Contributions

Hui Ma: Conceptualization (equal); investigation (equal); methodology (equal); project administration (equal); resources (equal); visualization (equal); writing – original draft (equal); writing – review and editing (equal). Yue Chen: Investigation (equal); methodology (equal); writing – original draft (equal). Xinzhou Deng: Investigation (equal); methodology (equal); writing – original draft (equal). Xuan Fang: Investigation (equal); methodology (equal); writing – original draft (equal). Wenhui Xiao: Investigation (equal); methodology (equal). Qian Long: Investigation (equal); methodology (equal); writing – original draft (equal). Lu Du: Investigation (equal); methodology (equal). Fuze Du: Investigation (equal); methodology (equal). Haonan Chen: Investigation (equal); methodology (equal). Yan Wang: Project administration (equal); resources (equal); visualization (equal); writing – review and editing (equal). Bo Gao: Project administration (equal); resources (equal); visualization (equal); writing – review and editing (equal). Xin Qiao: Conceptualization (equal); project administration (equal); resources (equal); visualization (equal); writing – review and editing (equal).

## Funding

This work was supported by the National Natural Science Foundation of China (No. 82204360) and the Beijing Natural Science Foundation (7232348).

## Ethics Statement

All procedures were approved by the Institutional Animal Care and Use Committee of the Academy of Military Medical Sciences, Academy of Military Sciences (Approval No. JFALR2018098). All animal studies are reported in compliance with the ARRIVE guidelines.

## Consent

The authors have nothing to report.

## Conflicts of Interest

The authors declare no conflicts of interest.

## Supporting information


**Figure S1:** MOO remodels gut microbiota and enhances SCFA production to alleviate PSD‐like behaviors. (A) Chemical structure of Morinda officinalis oligosaccharides (MOO). (B) Representative images of TTC‐stained brain slices from the Ctrl and MCAO‐24 h groups. Scale bars: 10 mm, *n* = 5. (C) Quantitative analysis of cerebral infarct volume, *n* = 5, ***p < 0.001. (D) Taxonomic composition analysis at the family and genus levels showing the relative abundance of microbial communities in Ctrl, PSD, and MOO groups, *n* = 8. (E) Additional alpha diversity indices (Chao1, Goods coverage, Simpson, Pielou's evenness, Faith's phylogenetic diversity, Shannon and observed species) in Ctrl, PSD, and MOO groups, *n* = 8. (F) Schematic diagram of the fecal microbiota transplantation (FMT) experimental design. (G) Quantitative analysis of time spent in the central area of the OFT, duration in the open arms of the EPM, and immobility time in the TST and FST following FMT, *n* = 8, *p < 0.05, **p < 0.01, ***p < 0.001. Data are presented as mean ± SEM. (H) Representative movement tracks and heat maps in the OFT and EPM; PSD: PSD model mice; MOO: PSD mice treated with MOO; FMT‐PSD: recipient PSD mice receiving fecal microbiota from PSD donors; FMT‐MOO: recipient PSD mice receiving fecal microbiota from MOO‐treated PSD donors.
**Figure S2:** MOO attenuates hippocampal inflammation, preserves blood–brain barrier integrity, and restores neuronal activity in PSD mice. (A) Evans blue stained images. (B) Quantification of evans blue stained assay, normalized to serum dye content and brain tissue weight. Significance was determined by one‐way ANOVA with Tukey's post hoc test, *n* = 4, **p < 0.01, ***p < 0.001. Ctrl, control; PSD, post‐stroke depression; MOO, morinda officinalis oligosaccharides. (C) The bar graph shows the significant differences in the concentration of TNF‐α, IL‐6, and LPS in hippocampal homogenates of each group. Significance was determined by one‐way ANOVA with Tukey's post hoc test, *n* = 5, **p < 0.01, ***p < 0.001. Data are presented as mean ± SEM. (D) Representative immunofluorescence staining images of c‐Fos (green) co‐labeled with DAPI (blue) in the hippocampus. (E) Quantification of c‐Fos^+^ cells in the hippocampal region of Ctrl, PSD, and MOO groups, *n* = 6, *p < 0.05, ***p < 0.001. Data are presented as mean ± SEM. (F) Representative immunofluorescence staining images of CaMKIIα (red) and c‐Fos (green) in the hippocampus.
**Figure S3:** Hippocampal proteomic profiling and subcellular localization of DEPs. Heatmap of differentially expressed proteins between PSD and Ctrl groups (labeled by gene names). (B) Heatmap of differentially expressed proteins between MOO and PSD groups (labeled by gene names).Schematic diagram of proteomic analysis, *n* = 3. (C) Subcellular localization of DEPs between PSD and Ctrl groups. (D) Subcellular localization of DEPs between MOO and PSD groups.
**Figure S4:** Hippocampal proteomic profiling and subcellular localization of DEPs. (A) Venn diagram depicting the overlap between the DEPs which up‐regulated in the PSD group and down‐regulated in the MOO group. (B) Genomes (KEGG) pathway enrichment analysis of overlapping DEPs. (C) Sankey chart of overlapping DEPs. (D) Venn diagram depicting the overlap between the DEPs which down‐regulated in the PSD group and up‐regulated in the MOO group. (E) Genomes (KEGG) pathway enrichment analysis of overlapping DEPs. (F) Sankey chart of overlapping DEPs.
**Figure S5:** Butyrate supplementation significantly restored PSD‐induced colonic injury. Representative immunofluorescence images of Claudin1, ZO‐1 and Occludin. Merged images with DAPI staining show protein expression in the Ctrl, PSD, and Buty groups. Ctrl, control; PSD, post‐stroke depression; SB, Butyric acid.

## Data Availability

The mass spectrometry proteomics data have been deposited in the ProteomeXchange Consortium via the PRIDE partner repository under accession number PXD081019. The raw 16S rRNA sequencing reads have been deposited in the NCBI Sequence Read Archive (SRA) under BioProject accession number PRJNA1531781. Other data supporting the findings of this study are available from the corresponding author upon reasonable request.
