# Supplementary material for: Morinda Officinalis Oligosaccharides Alleviate Post‐Stroke Depression‐Like Behaviors Alongside Increased Gut Microbial Diversity and Butyrate Levels and Reduced Hippocampal Pla2g4a Expression
Source: CNS Neurosci Ther. 2026 Oct 30;32(11):e71238. doi: 10.1002/cns.71238 (PMC13720668; doi:10.1002/cns.71238)
Supplement: Supplementary file 1 [file CNS-32-e71238-s001.docx]

**Supplementary Fig. 1**

**MOO remodels gut microbiota and enhances SCFA production to alleviate PSD-like behaviors.**

(A)Chemical structure of Morinda officinalis oligosaccharides (MOO). (B) Representative images of TTC-stained brain slices from the Ctrl and MCAO-24h groups. Scale bars: 10 mm, n=5. (C) Quantitative analysis of cerebral infarct volume, n=5, ***p < 0.001. (D) Taxonomic composition analysis at the family and genus levels showing the relative abundance of microbial communities in Ctrl, PSD, and MOO groups, n=8. (E) Additional alpha diversity indices (Chao1, Goods coverage, Simpson, Pielou's evenness, Faith's phylogenetic diversity, Shannon and observed species) in Ctrl, PSD, and MOO groups, n=8. (F) Schematic diagram of the fecal microbiota transplantation (FMT) experimental design. (G) Quantitative analysis of time spent in the central area of the OFT, duration in the open arms of the EPM, and immobility time in the TST and FST following FMT, n=8, *p < 0.05, **p < 0.01, ***p < 0.001. Data are presented as mean ± SEM. (H) Representative movement tracks and heat maps in the OFT and EPM; PSD: PSD model mice; MOO: PSD mice treated with MOO; FMT-PSD: recipient PSD mice receiving fecal microbiota from PSD donors; FMT-MOO: recipient PSD mice receiving fecal microbiota from MOO-treated PSD donors.

**Supplementary Fig. 2**

**MOO attenuates hippocampal inflammation, preserves blood–brain barrier integrity, and restores neuronal activity in PSD mice.**

(A) Evans blue stained images. (B) Quantification of evans blue stained assay, normalized to serum dye content and brain tissue weight. Significance was determined by one-way ANOVA with Tukey's post hoc test, n = 4, **p < 0.01, ***p < 0.001. Ctrl, control; PSD, post-stroke depression; MOO, morinda officinalis oligosaccharides. (C) The bar graph shows the significant differences in the concentration of TNF-α, IL-6, and LPS in hippocampal homogenates of each group. Significance was determined by one-way ANOVA with Tukey's post hoc test, n = 5, **p < 0.01, ***p < 0.001. Data are presented as mean ± SEM. (D) Representative immunofluorescence staining images of c-Fos (green) co-labelled with DAPI (blue) in the hippocampus. (E) Quantification of c-Fos^+^ cells in the hippocampal region of Ctrl, PSD, and MOO groups, n = 6, *p < 0.05, ***p < 0.001. Data are presented as mean ± SEM. (F) Representative immunofluorescence staining images of CaMKⅡα (red) and c-Fos (green) in the hippocampus.

**Supplementary Fig. 3**

**Hippocampal proteomic profiling and subcellular localization of DEPs.**

(A) Heatmap of differentially expressed proteins between PSD and Ctrl groups (labeled by gene names). (B) Heatmap of differentially expressed proteins between MOO and PSD groups (labeled by gene names).Schematic diagram of proteomic analysis, n=3. (C) Subcellular localization of DEPs between PSD and Ctrl groups. (D) Subcellular localization of DEPs between MOO and PSD groups.

**Supplementary Fig. 4**

**Hippocampal proteomic profiling and subcellular localization of DEPs.**

(A) Venn diagram depicting the overlap between the DEPs which up-regulated in the PSD group and down-regulated in the MOO group. (B) Genomes (KEGG) pathway enrichment analysis of overlapping DEPs. (C) Sankey chart of overlapping DEPs. (D) Venn diagram depicting the overlap between the DEPs which down-regulated in the PSD group and up-regulated in the MOO group. (E) Genomes (KEGG) pathway enrichment analysis of overlapping DEPs. (F) Sankey chart of overlapping DEPs.

**Supplementary Fig. 5**

**Butyrate supplementation significantly restored PSD-induced colonic injury.**

Representative immunofluorescence images of Claudin1, ZO-1 and Occludin. Merged images with DAPI staining show protein expression in the Ctrl, PSD, and Buty groups. Ctrl, control; PSD, post-stroke depression; SB, Butyric acid.
